# Dr. Alfred M. Haight

**Published:** 1912-09

**Authors:** 


Dr. Alfred M. Haight of White Plains, N. Y., a graduate of the N. Y. Homoeopathic Medical College and Hospital, 1879, died suddenly of heart disease at Ocean Grove, N. J., July 14, aged 56. He was a member of the American Institute of Homoeopathy and of the State and County organization and was attending physician to the White Plains Hospital and Jennie Clarkson Orphans' Home.

